# Eave tubes for malaria control in Africa: Videographic observations of mosquito behaviour in Tanzania with a simple and rugged video surveillance system

**Published:** 2017-07-01

**Authors:** Sergej Sperling, Michael Cordel, Scott Gordon, Bart G.J. Knols, Andreas Rose

**Affiliations:** 1 Biogents AG, Weißenburgstraße 22, 93055 Regensburg, Germany; 2 Radboud University Nijmegen, Department of Environmental Science, Heyendaalseweg 135, 6525 AJ, Nijmegen, The Netherlands

## Abstract

**Background:**

Eave tubes are novel mosquito control devices that help to protect households against malaria vectors and other mosquitoes. They are installed in the upper walls of human habitations after the eaves have been closed. Mosquitoes trying to enter through these tubes are intercepted by electrostatic netting that can be treated with a variety of insecticides. Using video, mosquito behaviour and duration of contact with netting in eave tubes was recorded and analysed to assess contamination with insecticides under semi-field and field conditions.

**Materials and methods:**

Off-the-shelf action cameras were used to observe behaviour of mosquitoes in eave tubes near Ifakara, Tanzania. In an experimental hut in a screen house, we observed *Anopheles arabiensis* females on electrostatic eave tube netting treated with bendiocarb powder or with *Beauveria bassiana* spores, both in comparison to untreated netting. In village houses that had been equipped with eave tubes we observed the behaviour of wild mosquitoes towards electrostatic netting treated with bendiocarb. Results were evaluated using a short-contact assay (5 second exposure).

**Results:**

In the semi-field setup, the median contact time of *An. arabiensis* on bendiocarb-powdered eave tube nets was 276.4 sec (n=56), compared to 26.3 sec on the control (n=59). Of all the mosquitoes observed on the treated net, 94.6% had contact times of more than 5 seconds on the bendiocarb-powdered netting. The median time on nets powdered with *B. bassiana* spores was 34.4 sec (n=26), compared 37.1 sec in the untreated control (n=22). 88.5% of the mosquitoes spent more than 5 seconds on the treated nets. In the field we recorded 106 individual mosquitoes of unknown species inside tubes. They spent a median time of 70.9 sec on the bendiocarb-treated netting, with 90.6% remaining there for more than 5 seconds.

**Conclusions:**

We have found no indication that the behaviour of mosquitoes on electrostatic eave tube netting, treated either with bendiocarb powder or with *B. bassiana* spores, interferes with successful transfer of lethal doses of these insecticidal actives. The videographic set-up used in this study is simple, sturdy and reliable enough to observe and analyse mosquito behaviour under field conditions.

## 1 Introduction

The fight against malaria has made historic gains across sub-Saharan Africa over the past fifteen years. In countries where the use of insecticide-treated mosquito nets (ITNs), indoor residual spraying (IRS), improved diagnostic tests, and highly effective antimalarial drugs have been scaled up, mortality rates in children under five years of age have fallen markedly [1]. These reductions have been achieved mainly through vector control measures involving the widespread distribution of ITNs and indoor residual spraying (IRS) with insecticides [2]. Currently, ITNs and the majority of IRS campaigns predominantly rely on pyrethroids. Unfortunately, resistance to pyrethroids has emerged in anopheline mosquitoes across Africa and looms as a major threat to vector control. Resistance to pyrethroids, which was rare just fifteen years ago, has now become so severe that no African country has fully pyrethroid susceptible malaria vector populations [3]. Resistance to the alternative insecticides approved for public health use, such as organophosphates, carbamates and organochlorines, has also been reported in the principal malaria vector *Anopheles gambiae s.l.* [4–6]. To combat this trend, research is needed to develop new vector control tools that do not contribute to the growing resistance problem.

Improvements in house construction such as screening of doors and windows, closing the eaves, filling of cracks in walls, and installing ceilings are all related interventions that help decrease contact between malaria vectors and humans, and therefore help reduce malaria transmission without the use of pesticides [7]. *Anopheles gambiae s.l.*, one of the major African malaria vectors, locates its hosts by following odour plumes close to the ground and flying upwards when a vertical surface is reached. Open eaves then serve as the entry point for mosquitoes into the house. As a consequence, closure of the eaves has been shown to reduce house entry by malaria vectors and therefore transmission of disease [8–10].

Andriessen *et al.* [11] described a novel method of exposing mosquitoes to insecticides by using mosquito netting treated with a coating that binds powder formulations of insecticides through electrostatic forces. Using standard WHO bioassays they exposed six pyrethroid-resistant strains of *Anopheles* from across Africa to a concentration of deltamethrin on electrostatic netting similar to or lower than that on a long-lasting deltamethrin-coated bednet. Significantly higher mortality occurred in mosquitoes exposed to the electrostatic net, effectively breaking resistance. The uptake of insecticidal particles from electrostatic netting is much more efficient than the uptake from a long-lasting insecticide-treated bednet at similar or much-reduced target doses of active ingredient per unit surface area. This increased bioavailability of active ingredients may provide a means to lower the total amount of insecticide needed for effective vector control.

Eave tubes combine the concepts of mosquito proofing human habitation, and attracting mosquitoes to an insecticidal device using human odours from inside the house as attractants. They are essentially 6-inch PVC pipes installed in the walls of houses in the closed space that normally represents the eave [12,13]. Once inside the tube, mosquitoes encounter an insecticidal net that blocks their entry. The net is electrostatically charged and can bind a variety, or even mixtures of powdered insecticides. This exposure method has been shown to be highly effective and can break pyrethroid resistance. Compared to deltamethrin-coated bednets (Permanet 2.0), electrostatic nets with powdered deltamethrin at a 15-fold lower dose and an exposure time of only 5 seconds still induced a high mortality even in highly pyrethroid-resistant mosquitoes [11]. These electrostatic nets are also a promising method for transferring spores of entomopathogenic fungi, such as *Beauveria bassiana*, to mosquitoes [14,15].

Although low doses and short exposure times were shown to be sufficient for an effective transfer of lethal doses of insecticidal particles under laboratory conditions [11], it remained unknown if the natural behaviour of mosquitoes in eave tubes met these minimum conditions. The behaviour and contact times of mosquitoes to electrostatic netting in eave tubes under conditions that resemble the field as much as possible thus needed to be described and quantified. We therefore set out to document mosquito behaviour in eave tubes using digital video recordings, first in a semi-field screen house similar to the one described by Ferguson *et al.* [16] and thereafter in a village setting in rural Tanzania.

## 2 Materials and methods

### 2.1 Video system

**Figure 1:**
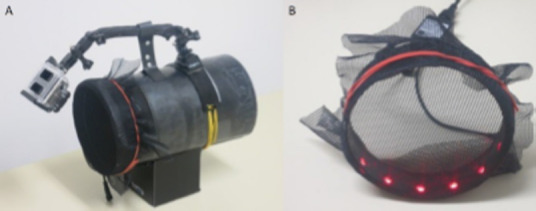
A: Prototype eave tube, with mounted camera, red LED light source and electrostatic netting. B: Detailed view of the red LED light source with electrostatic netting, which can be attached to an eave tube.

Video observations of mosquitoes entering eave tubes and making contact with electrostatic netting were performed using a rugged and easy to use system made from off-the-shelf action cameras and red LEDs as light sources (Figure 1). The cameras (GoPro® Hero3+ Black Edition, GoPro Inc., San Mateo, USA), in their dust- and waterproof plastic casings, were mounted using the provided camera mounts and/or additional mounting equipment, at a distance of approximately 5 cm from the netting. Depending on the available space around the tube, the cameras were aimed at the tubes at different angles. Each camera was fitted with a 64GB SD Card (SanDisk Extreme or Ultra, micro SDXC UHS-I Card, SanDisk Corporation, Milpitas, California, USA). External battery packs (capacity: 12000 mAh; max output Voltage 2 V, Logilink, 2direct GmbH, Germany) provided energy through USB extension cords. Set-up and control was performed using the GoPro App Version 2.4 via Android-based tablets and mobile phones through WiFi. The cameras were set on video mode, with a resolution of 1080p, medium angle, ‘Auto Low Light’ on, a frame rate of 48fps, and WiFi on.

The red LED light sources were made from 20 cm pieces of an LED strip (LED-Strip RGB, 5m, Müller-Licht International GmbH, Germany), each with 6 LEDs. The strips were mounted on the inner end of the eave tubes using self-made plastic belts. 12 V batteries (locally purchased motorcycle batteries) served as power supplies. The LED colour ‘red’ (wavelength: 635 nm) was chosen, because it was reported not to be an attractant for anopheline mosquitoes [17,18]. Although *Anopheles gambiae* may see red light of the wavelength we used [19], it probably did not affect the attractiveness of the eave tubes, for it has been shown that mosquitoes in search of a host mainly use olfactory cues and only when in close proximity of the host will use other cues to locate a landing site [20,21].

### 2.2 Semi-field studies

**Figure 2:**
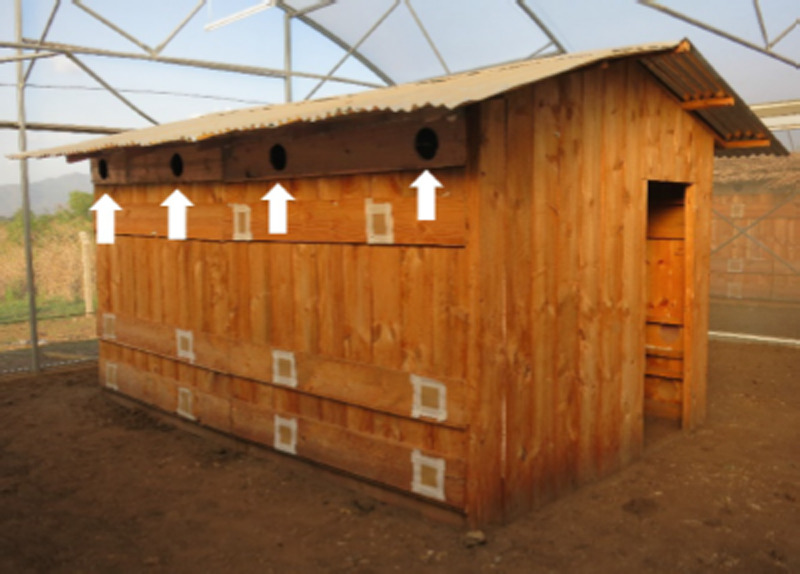
Semi-field experimental wooden hut used for the video surveillance experiments in a screen house in Kining’ina, Ifakara, Tanzania. Eave tubes are indicated by white arrows.

**Figure 3:**
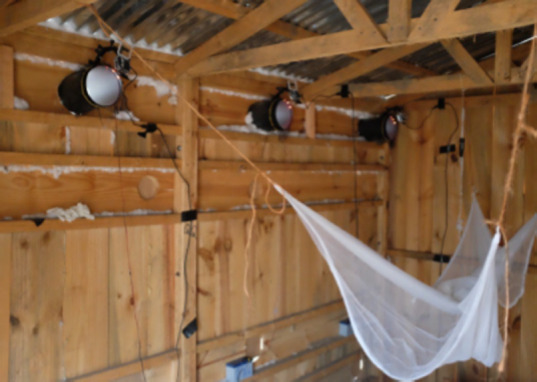
Inside view of the experimental hut showing three eave tubes equipped with cameras and red LED lights. Slits and other openings were closed with cotton wool.

Semi-field experiments were performed in November 2014 and February/March 2015 in a compartment of a screenhouse in Kining’ina, near Ifakara, Kilombero Valley, Morogoro Region, Tanzania. The dimensions of this compartment were 10 x 10 x 4 m (L x W x H). It contained a wooden experimental hut (dimensions: 4.2 x 2.6 x 2.5 m, Figure 2), which had a door, no windows, and a roof made from corrugated iron sheets. Slits and holes were sealed with cotton wool or jute to eliminate additional points of mosquito entry. Eight eave tubes (PVC-Tubes, outer Ø 15.24 cm, inner Ø 15.00 cm) were installed at 1.9 m height, which was found to be the height at which most mosquitoes were attracted into tubes [12], at 1 m distance from each other. Six of the tubes were fitted with video cameras (Figure 3), the other two were sealed. No vegetation was present in the compartment.

Two trials were performed in the semi-field system, comparing two treatments. Trial 1 compared the behaviour of *An. arabiensis* towards electrostatic netting treated with bendiocarb 1.25% (Ficam® D, Bayer AG, Leverkusen, Germany) to untreated electrostatic netting and was performed in November 2014 and February 2015. Trial 2 was similar but used spores of the entomopathogenic fungus *Beauveria bassiana* (Laverlam International, Butte, Montana, USA) and was conducted in March 2015. For the application procedure of bendiocarb or *Beauveria* spores on electrostatic netting we refer to the procedures described by Snetselaar *et al.* [22]. The netting in eave tubes without a camera was left untreated. Treatment and control nettings were exchanged alternately each night. After removing bendiocarb-treated nettings, the eave tubes were rinsed with water to prevent contamination during the control experiments. Light mounts were assigned to specific treatments as an additional measure to prevent contamination.

A volunteer sleeping in a bed inside the experimental hut for the entire night served as the source for host cues. Experiments were performed using *An. arabiensis* from a colony reared at the Ifakara Health Institute (IHI). The strain originated from wild specimens from the Kilombero valley around Ifakara, where *An. arabiensis* has become the most important malaria vector [23]. The colony was maintained under semi-field conditions. Larvae were fed with ground TetraMin® fish food (Tetra GmbH, Melle, Germany), the adults provided with glucose water (10%) and blood meals from the arms of human volunteers. Every evening between 19.00 and 19.30 hrs and prior to the experiments, 200-300 females, 3-7 days old, were released inside the compartment. The mosquitoes were starved for 6 hours prior to their release.

The highest biting activity of *An. arabiensis* is reported to be after sunset, in the first half of the night [24,25]. Video recordings therefore started after sunset, immediately after the release of the mosquitoes and with the volunteer occupying the experimental hut. The cameras then filmed for approximately 4.5 hours, until the SD-card was full. Experiments were repeated until contacts of at least 20-30 individual mosquitoes were observed for each treatment.

### 2.3 Field studies

**Figure 4:**
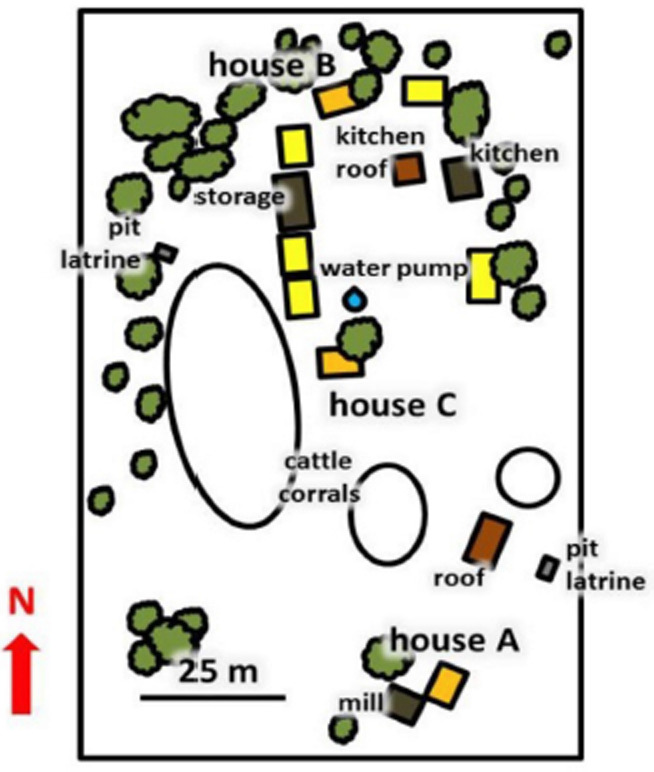
Layout of the compound in Sagamaganga village in southern Tanzania where the field trials were performed. Houses that served as sleeping quarters are shown in yellow or orange. Videography was performed in houses A, B, and C (orange).

Field studies were performed between March and the end of July 2015, in Sagamaganga, 23 km from Ifakara, in a farming compound consisting of nine human habitations around a central square (Figure 4). Rice fields and some grazing lands surrounded the compound. There were also cattle and goats that spent the nights in two of three corrals near the human habitations. The complete area of the compound covered approximately 7,800 m^2^. The houses were made of bricks, had corrugated iron roofs and typically consisted of two rooms; one that was used as sleeping quarters for 3-6 people, while the other mainly served as a storage room. The houses had one door and three to four windows. All beds in the houses had bed nets. A doorway with a loose curtain connected the two rooms of each house; house A had an additional open space of about 20 cm between the roof and the dividing wall. This means that an exchange of host odours and mosquitoes was easily possible between the two rooms.

**Figure 5:**
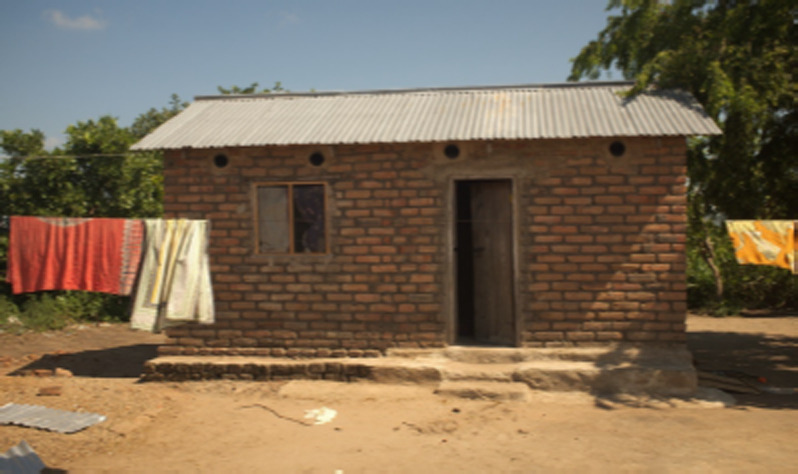
Front view of house B after installation of the eave tubes. The bedroom is on the left.

In each of the nine houses of the compound that served as sleeping quarters, eaves and the gaps around window and doorframes were closed with cement and mosquito netting was fixed on the window frames. Eight eave tubes were built into each house, four in the front and four in the back (Figure 5). Thus, four eave tubes were present in the bedroom and four in the other room. All eave tubes were fitted with electrostatic netting treated with bendiocarb (Ficam® D, Bayer, Leverkusen, Germany) as described by Snetselaar *et al.* [22].

**Figure 6:**
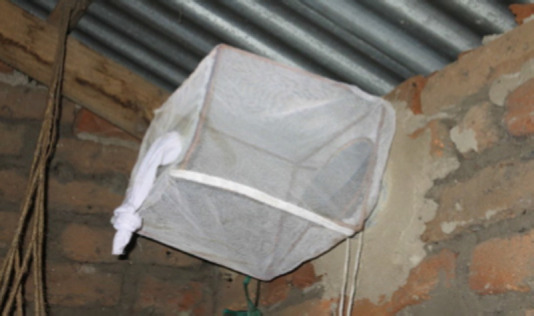
Eave tube entrance trap mounted on an eave tube in a house in Sagamaganga. The white net treated with Ficam W® to ensure rapid knockdown upon contact.

Three houses with a minimum distance of 40 meters between them were selected for the study (Figure 4). In the sleeping rooms of each of these houses, two eave tubes were observed using the video system described above. As in the semi-field, recordings began after sunset, between 19.00 and 19.30 hrs. The other two eave tubes in the room had no netting and instead had entrance traps fitted on the tubes (Figure 6). The netting of these entrance traps was treated with bendiocarb wettable powder (Ficam® W, Bayer AG, Leverkusen, Germany) to ensure rapid knockdown of mosquitoes that entered in the cages through the tubes. In addition, one CDC miniature light trap (Model 512, John W Hock Co., Gainesville, Florida, USA) was suspended in the late afternoon next to the bednet in each of the sleeping rooms at a height of about 1 m. Traps were run for 24 hrs and catches were collected and identified the following afternoon. Experiments were conducted for two nights each week.

### 2.4 Analysis of video data

Initial observations of video recordings showed that it was possible to distinguish between mosquitoes flying above the electrostatic netting of the eave tubes and those actually making contact with it, either through briefly touching it or by landing on its surface. These behaviours could be monitored and quantified using the software Ethovision XT 11 (Noldus AG, Wageningen, The Netherlands) and Excel (Microsoft Office 2013, Microsoft Corp., Redmont, Washington, USA). We distinguished three behaviour patterns: ‘flying’, ‘bouncing’ and ‘sitting’. Our definition of a ‘sitting’ mosquito also included specimens that were walking on the surface, a behaviour that was observed only rarely. Due to the relatively short distance between the camera and the netting, the camera would also pick up wing or leg movements, which then resulted in velocity readings of up to 10 cm/sec. ‘Bouncing’ and ‘flying’ resulted in even larger velocity readings (up to 100 cm/sec) and were thus distinguishable from ‘sitting’. Thus ‘sitting’ was defined as moving very slowly (velocity 1-10 cm/sec) or not moving at all (0-1 cm/sec). ‘Flying’ was defined as moving with a high velocity without touching the surface of the netting (>10 cm/sec) and a ‘bouncing’ mosquito showed sitting and flying behaviour in short sequences. The periods during which an individual mosquito was ‘sitting’ were summed up to determine the total minimum contact time. Although the camera resolution is sufficient to analyse the behaviour with Ethovision XT, it was impossible to distinguish different mosquito species from the footage in the field where a variety of species abound.

The cameras generated 4.5 hrs of video recordings every night. The firmware of the camera divided the recordings in video sequences with a length of approximately 17 min in Mp4-format. Every recording was checked manually to identify sequences in which mosquitoes entered tubes and made contact with the netting. For the experiments, the sections of each video with single mosquito contacts (if two or more mosquitoes were moving simultaneously, the sequences were discarded because these could not be analysed by the software) were cut and converted to avi format using the program Freemake Video Converter (Version 4.1.5.4, Ellora Assets Corporation, USA). The videos were subsequently analysed by the software Ethovision XT 11 (Noldus AG, Wageningen, The Netherlands) using the following parameters/settings:

- Arena: The experimental area was defined as the electrostatic netting fitted over the eave tube. The software offers to make so-called exclusions to the defined arena. Exclusions are areas artificially excluded from the analysis due to their potential to interrupt the tracking procedure. This feature was used to exclude back lights (LEDs of the ring which directly hit the camera) or mosquitoes sitting on the netting inside the hut.
- Software: Centre point detection, sample rate: 16 frames/sec (data collection every 0.21 sec); method: dynamic subtraction, subject colour: brighter (12-255), Frame weight: 10; contour erosion: 1 pixel; Contour dilation: 10 pixels, subject size: 200-125000.
- Output: Time-based coordinates (x and y) of the subject (mosquito), resulting in the distance moved within every 0.21 sec. This distance was then used to calculate the velocity in cm/sec.
- Alignment: The cameras were set up at an angle; therefore the arena in the video appeared as an ellipsoid. To generate the correct velocity data, the aspect ratio of the ellipsoid was used as the correction factor to calculate the time-based coordinates.

**Figure 7:**
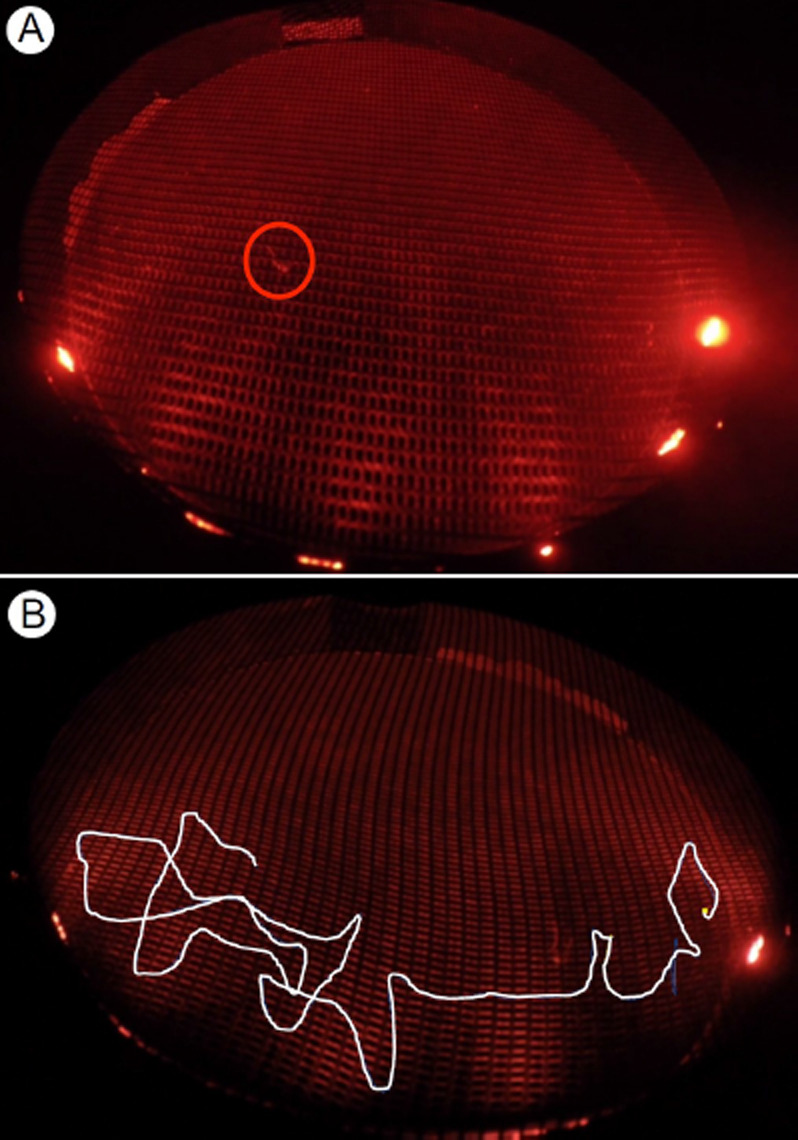
A. Screenshot from a video recording of a mosquito making contact with bendiocarb-treated netting. B: Visualised mosquito track on electrostatic netting after Ethovision XT analysis.

Contact time was analysed by calculating the motion speed of the mosquito. The time spent by the mosquito moving at a velocity of 10 cm/sec or less was summed up; velocity is the distance moved in 0.21 seconds. This timeframe is a fixed time unit and default setting by Ethovision and is not changeable. If therefore a mosquito was moving at a velocity of 2 cm/sec or less per 0.21 seconds timeframe it would be considered as motionless and in contact with the netting, even though wing or leg movements were interpreted by the software as movements of the whole mosquito (spatial change of the centre point) with velocity readings up to 10cm/sec. A mosquito as observed in the video footage and the resulting track are shown in Figure 7a and 7b, respectively.

The behaviour was analysed with the calculated velocity readings used for the analysis for contact times by a computational algorithm developed in the programming language Visual Basic for Applications (VBA 7.0, Microsoft Cooperations, Redmond, USA).

Operation 1: The first step was to assign ‘1’ or ‘0’ to different velocity readings. A ‘1’ was assigned to velocities ≥10cm/sec (status: no contact with the netting) and ‘0’ to all velocities <10cm/sec (status: in contact with the netting, referred to the analysis of the contact time). Result: ‘1 -0’-sequence for contact (Sequence SCO).

Operation 2: Each ‘1’ or ‘0’ information from the sequence SCO was analysed according to their numerical surrounding area to define behavioural changes in modus ‘contact with netting’ and ‘no contact with netting’ by subtracting the previous data point, getting a new ‘1-0’-sequence for changes (Sequence SCH; 1=change; 0= no change). In a behavioural context, a mosquito changes its behaviour from ‘sitting’ to ‘flying’ or vice versa (single change). More frequent changes (a long sequence alternating between ‘0’ and ‘1’) were defined as ‘bouncing’.

Operation 3: To distinguish between single changes and multiple changes (‘bouncing’) we combined the sequence SCO and SCH in a further mathematical step [equation: a= (x_SCo_+y_SCh_)*5], to obtain different numbers for each event (for bouncing events =1 or 6, for sitting=5 and for flying=0). The referred time sequences for each event were summed up to get the total time mosquitoes spent showing each behavioural pattern.

Mann-Whitney U tests were used to quantify differences of single mosquito contact times between treatments. Prior to statistical analysis the behaviour data (proportions of time spent showing a specific behavioural pattern) was arcsine transformed. The statistical software PAST 3.0 (freeware, Sweden) was used to calculate statistical differences [26].

### 2.5 Ethics

For the light trap catches and household surveys written informed consent was obtained in the appropriate language from an adult household member. The same applied to technical assistants that volunteered in blood feeding of mosquitoes that were used in the semi-field experiments. Approval from local leaders in Sagamaganga village was obtained before commencing trapping and filming activities.

## 3 Results

### 3.1 Semi-field trials

**Figure 8:**
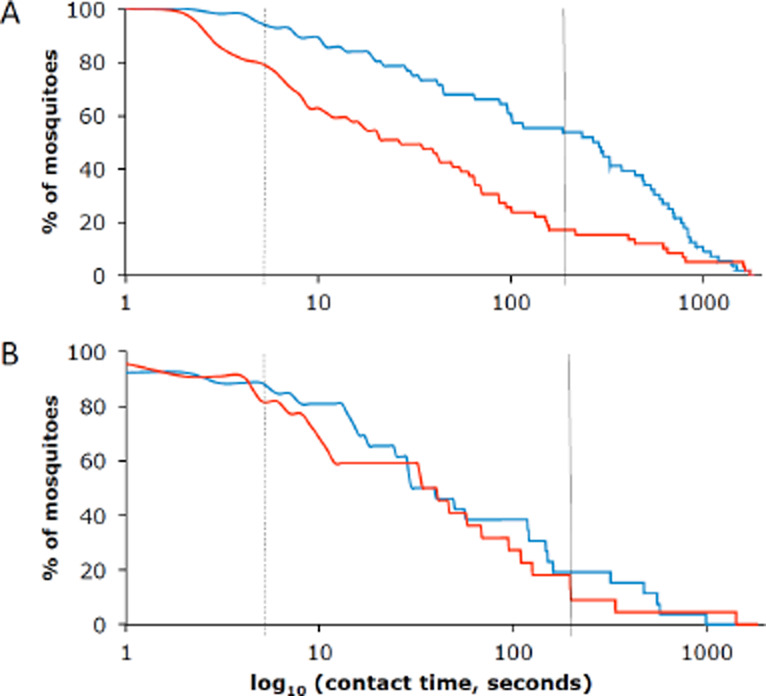
A: Percentage of mosquitoes on the bendiocarb-treated (blue) and untreated (red) nets over time (log scale), with the dashed line at 5 seconds and the solid line at 180 seconds. B: Same, but for netting treated with B. bassiana (blue) and untreated netting (red).

**Figure 9:**
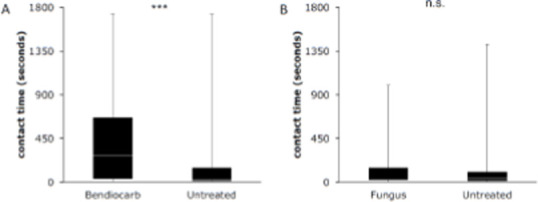
Boxplot of the contact times of An. arabiensis females on (A) bendiocarb-powdered (Ficam® D) and on untreated electrostatic netting and (B) on dusted B. bassiana fungal spores and on untreated electrostatic netting.

In the semi-field system with a wooden hut and a human volunteer protected by a bednet, we exclusively used laboratory-reared females of the most common malaria vector in the Kilombero Valley, *An. arabiensis* [23]. Of 56 individual mosquitoes that were video recorded in the eave tubes, 94.6% spent more than 5 seconds on the bendiocarb -powdered netting. In comparison, only 79.7% of 59 mosquitoes that were filmed on netting that was not treated spent more than 5 seconds on it (Figure 8a). Half (53.6%) of the mosquitoes spent the standard WHO exposure time for insecticide bioassays of 180 seconds or longer on the bendiocarb-treated netting. This proportion was substantially lower for the fungus treatment (19.2%; Figure 8b). The difference in contact times between bendiocarb-powdered electrostatic netting (mean: 397.2 sec, median: 276.4 sec) and untreated netting (mean: 181.3 sec, median: 26.3 sec; Figure 9a) was highly significant (p<0.001). In contrast to the trials with bendiocarb, there was no significant difference in the contact times between electrostatic netting treated with *B. bassiana* spores (mean: 151.6 sec, median: 34.4 sec) and untreated netting (128.0 sec; median: 37.1 sec; Figure 9b).

**Figure 10:**
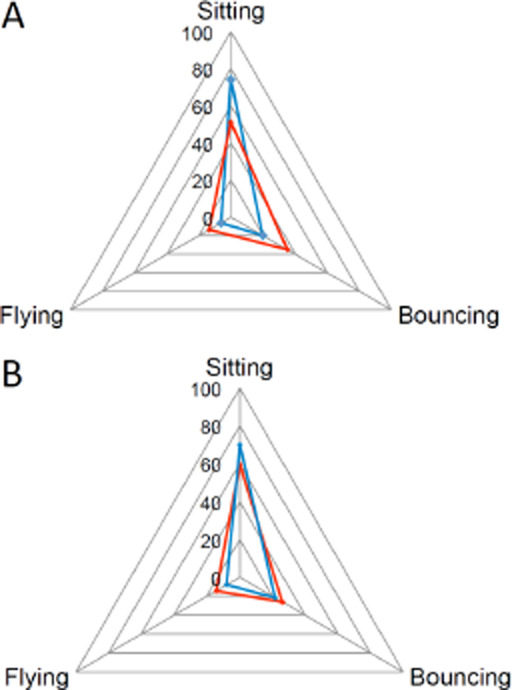
Radar chart of the three behaviour patterns (“sitting”, “flying”, “bouncing”) observed in female An. arabiensis entering the eave tubes in the experimental hut and their proportion for (A) bendiocarb-powdered (blue, N=56) and untreated (red, N=59) netting and (B) for B. bassiana-powdered (blue) and untreated (red) netting.

The mosquitoes also showed proportionally more ‘sitting’ and less ‘bouncing’ and ‘flying’ behaviour when confronted with electrostatic netting powdered with bendi-ocarb, compared to untreated netting (Figure 10a). In contrast, when confronted with electrostatic nets powdered with spores, the mosquitoes showed similar proportions of time ‘sitting’, ‘bouncing’ and ‘flying’ behaviour, compared to the untreated control (Figure 10b).

### 3.2 Field trials

In the field, we were able to record and distinguish 106 single mosquito tracks with the video surveillance system. These 106 wild mosquitoes of unknown species identity spent an average of 169.1 sec (median=70.9 sec) on the bendiocarb-powdered eave tube netting, with 90.6% (96/106) having contact times of longer than 5 seconds.

Mosquito collections from eave tube traps fitted with bendiocarb-treated netting and CDC miniature light traps next to occupied bednets were compared on 30 days. Both collection methods captured the same species: *An. gambiae s.l*., *An. funestus s.l., An. pharaoensis*, *An. squamosus*, *An. coustani s.l.*, *Mansonia spp.*, *Aedes aegypti and Culex spp.* The proportion of malaria vectors caught in indoor CDC miniature light traps (56.9%) was higher compared to the eave tube entrance traps (32.6%). The most abundant mosquito found in the CDC traps was *An. gambiae s.l.* (31.2%), compared to 16.0% in the eave tube entrance traps. *Culex spp.* constituted the majority of mosquitoes collected in the eave tube traps (58.9%), compared to 26.6% in the CDC traps (Table 1). In total, the indoor CDC light traps caught 3450 mosquitoes, more than tenfold the number collected in the eave tube traps (319).

**Table 1:** Mosquito species composition of the catches in eave tube entry traps and CDC miniature light traps in Sagamaganga, from 30 sampling days between the 25.03.2015 and the 28.07.2015, with two sampling days per week. Total of all mosquitoes collected in houses A, B, and C (Figure 4).

	Eave tube entry traps		CDC light trap	
Mosquito species	n	%	n	%
Malaria vectors	77	24.1	1561	45.2
*An. funestus s.l.*	26	8.2	486	14.1
*An. gambiae s.l.*	51	16.0	1075	31.2
Other mosquitoes	242	75.9	1889	54.8
*An. squamosus*	3	0.9	28	0.8
*An. pharaoensis*	24	7.5	374	10.8
*An. coustani s.l.*	14	4.4	318	9.2
*Culex spp.*	191	59.9	919	26.6
*Mansonia spp.*	8	2.5	248	7.2
*Aedes aegypti*	2	0.6	2	0.1

## 4 Discussion

A central criterion for the effectiveness of the eave tube and electrostatic net concept is that the contact times of mosquitoes on the electrostatic netting are long enough for the effective transfer of lethal doses of insecticide. Recent experiments with bendiocarb dust (Ficam® D, 1.25%w/w bendiocarb) on electrostatic netting showed that exposure times as short as 5 seconds induced 100% mortality in susceptible *Aedes aegypti* and *Culex quinquefasciatus* [11]. The authors used fluorescent insecticide powder on electrostatic netting and were able to detect powder particles on mosquitoes after as short as a 5 seconds of contact with the netting. They argue that in powder form the insecticide uptake by the mosquito is a lot higher than from impregnated materials (LLINs), where the insecticide is impregnated in the fibers and the effective (surface) concentration of insecticide is therefore much lower. With this improved bioavailability the authors were able to kill deltamethrin-resistant mosquitoes with deltamethrin powder on electrostatic netting when using only a fraction of the insecticide used in LLINs. The same insecticide product and electrostatic netting were used in the semi-field and field studies presented here. In an experimental hut in the semi-field, 94.6% of the 56 individual *An. arabiensis* that were analysed in video tracks spent more than 5 seconds under field conditions and in eave tubes that were installed in actual human habitations, 90.6% of the 106 individual mosquitoes had contact times of more than 5 seconds.

The WHO cone test is a standard bioassay test that was designed to assess the toxicity of materials to mosquitoes (e.g. LLINs). The standard exposure time, as suggested by WHO, is 180 seconds. A big disadvantage of this test is the close proximity of the mosquito to the tested/treated material. Since mosquitoes are more or less forced to have contact with the material this may result in an overestimation of the toxicity of a specific surface like an LLIN. Our experiments show a more realistic estimate of how long mosquitoes actually spent in contact with netting while trying to get into an occupied house. Especially the numbers from the field experiments (median of 71 sec) represent the most realistic case as there was a mixture of different culicine and anopheline species present and the data was collected over a course of 5 months, including the wet and the beginning of the dry season with the highest mosquito abundance.

**Figure 11:**
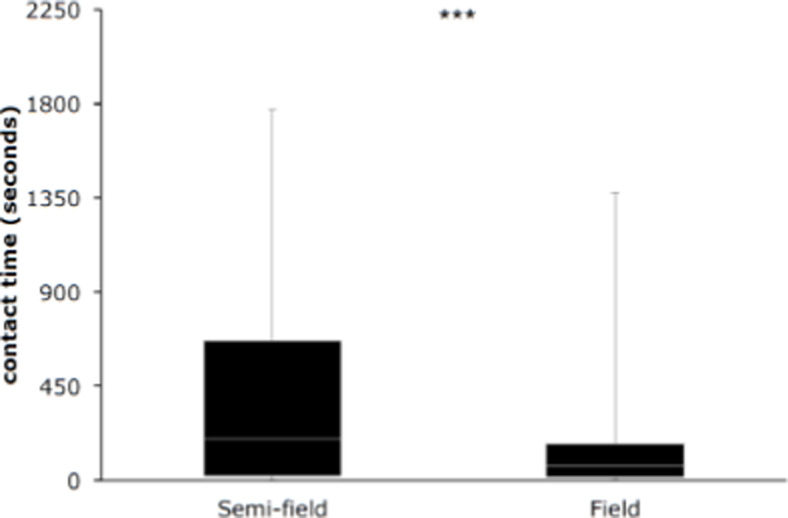
Boxplot of the contact times on electrostatic eave tube nets treated with bendiocarb for 59 observations of *An. arabiensis* females in the semi-field and 106 observations of undetermined mosquitoes in the field (Sagamaganga). Females of *An. arabiensis* in the semi-field setting spent significantly (p<0,001) more time on treated (mean: 406 sec; median: 199 sec) netting than wild mosquitoes in the field (mean: 169 sec; median: 71 sec).

Although the median contact times of the mosquitoes on the bendiocarb-treated netting were significantly longer in the semi-field (199 sec, N=106) than in the field (71 sec, N=59) (Figure 11), even the values observed in the field were still more than 14 times the time necessary for transfer of a lethal dose of insecticide from the electrostatic netting to the mosquito. When looking at the trap catches from the field study, these show a big proportion of *Culex* mosquitoes. As we did the semi-field work only with *An. arabiensis*, we assume that the difference in contact times possibly was caused by differences in behaviour of different mosquito species when these encountered the netting barrier during their host-seeing process.

The semi-field experiments with *An. arabiensis* revealed a marked difference between bendiocarb-dusted electrostatic netting and untreated control netting. The contact times for bendiocarb were significantly longer. Also, the proportion of mosquitoes observed ‘sitting’ on the netting with bendiocarb was significantly higher than that for the untreated control. Repellency of the bendiocarb formulation of any kind was therefore not observed, which is consistent with its classification as a contact insecticide. Although we did not quantify this, we observed that mosquitoes more frequently performed grooming behaviour when sitting on the bendiocarb-powdered netting, which could be in response to the transfer of insecticide particles to the insect.

In contrast, the ‘sitting’ behaviour on electrostatic eave tube nets powdered with *Beauveria* spores showed no significant difference to the untreated control. Infection rates with *B. bassiana* were very high (<95%) even with short exposure times and did not change even when comparing 5 seconds or 1 hour exposure to spores bound to electrostatic netting [14]. Out of 26 mosquitoes that were analysed 88.5% spent at least 5 seconds on the net powdered with spores, compared to 81.8% out of 22 mosquitoes on untreated netting. Even though the mosquitoes were not observed and checked for sporulation, it is safe to assume, that a very large proportion became infected by this bio-control agent during the trials [14]. One study even reported attraction of *An. stephensi* to caterpillars infected with *B. bassiana* and to oil formulation of spores on cloth [27]. It could be a specific reaction of *An. stephensi* or a synergistic effect of the oil formulation as in our study there was no apparent attractive effect of *B. bassiana* spores to *An. arabiensis*.

Since the field population of mosquitoes in Sagamaganga consists of several species, and culicine and anopheline mosquitoes could not be separated in the video recordings, entrance traps were attached to the eave tubes that were not under video surveillance in order to identify the species and number of individuals entering an eave trap. To monitor mosquito entrance into the houses despite the modifications, CDC miniature light traps were placed in the rooms, next to the bednet.

It is important to note that the numbers of mosquitoes collected in the eave tube traps are not representative for the total number of mosquitoes that may have tried to enter the houses through the eave tubes. The traps were installed in only two out of eight eave tubes in each house. Also, since the eave tube entrance trap is only a simple cage that is positioned over the inside end of the eave tube, it leaves a relatively large opening (the 6-inch tube itself) for mosquitoes to escape. It is therefore possible that mosquitoes left the trap before the knock-down effect of bendiocarb took effect, leading to an underestimation of the number of mosquitoes that actually entered the eave tubes where traps were installed. A netting lobster trap cone fitted inside the eave tube, as well as an additional insecticidal netting material loosely placed into the entrance trap could lead to more representative catches in future experiments. The video data presented here also do not give a reliable estimate of the actual number of mosquitoes entering the eave tube. They, too, were only applied on two of eight eave tubes, and due to a limited available memory size for the cameras, recordings were only performed for the first 4.5 hours of the scotophase, beginning at dusk, thus neglecting mosquitoes that entered the tubes after midnight. Also, we excluded footage with more than one visible mosquito in order to be able to measure contact times of individual mosquitoes only.

During the 30 nights in which these trials were performed, the six eave tube entrance traps collected 319 mosquitoes (Table 1). The most important malaria vectors made up 31.7% of the catch (101 mosquitoes), including 16.0% of *Anopheles gambiae s.l.*, 8.2% *An. funestus s.l.* and 7.5% *An. pharaoensis*. The largest proportion of mosquitoes was *Culex*, with 59.9%. In the same time period, the three indoor CDC miniature light traps collected a total of 3,450 mosquitoes, with malaria vectors making up 56.1% (1935 mosquitoes, with 31.2% *An. gambiae s.l.*, 14.1% *An. funestus s.l.* and 10.8% *An. pharaoensis*). This makes an average of 21.5 captured malaria vectors per night. The proportion of *Culex* was 26.6% of the complete catch.

Despite closing the eaves and screening the windows, indoor CDC light trap catches showed that many mosquitoes were still entering the houses. Over 30 days of sampling, eave tube traps collected only 5% as many malaria vectors as did the indoor CDC traps indicating other means of access are being utilized. Kirby *et al.* [10] showed that full screening of houses (windows, doors and ceilings) in The Gambia led to a 59% reduction in mean indoor vector density, while screening only the ceilings (similar to sealing the eaves) reduced the mean density by 49%. Njie *et al.* [28] demonstrated that closing the eaves resulted in a 66% reduction in the number of *An. gambiae s.l.* entering houses in which the eave gap is the major route of entry for mosquitoes. While structural improvements can certainly reduce human-vector contact, houses need to be relatively well built in the first place in order for screening efforts to be successful [29].

Lwetojiera *et al.* [23] analysed the species composition of *Anopheles* mosquitoes captured in two villages in distances of about 35 km (Idete) and about 50 km (Namawawala) to the field site in Sagamaganga over five consecutive years between 2008 and 2012. They too used CDC light traps next to occupied bed nets. Among the *An. gambiae s.l.,* the percentage of *An. arabiensis*, rose from 86% in 2008 (with 14% *An. gambiae s.s.*) to 100% in 2012. The situation is similar in Sagamaganga, with *An. arabiensis* now being the almost exclusive representative of the *An. gambiae s.l.* complex (Issa Lyimo, *pers. comm.*).

Due to ethical reasons, we performed the same modifications in every house of the compound. We could therefore not generate data on mosquito numbers that would have been captured with CDC miniature light traps in unmodified habitations.

Parker *et al.* [30] also used video equipment to observe mosquito behaviour on netting material that blocks the insects from reaching attractive cues, in their case ITNs occupied by sleeping volunteers. These authors described the mosquito behaviour similar to the classification used in the present study, using mosquito velocity to characterise the behaviour patterns ‘bouncing’, ‘resting’ and ‘swooping’ (i.e. not touching the surface of the netting, in our case ‘flying’). Since they observed the behaviour of mosquitoes towards a bednet, they had a much wider field of vision. Therefore they were able to pick a much smaller threshold to characterise the behaviour ‘resting’ (1.33 mm/sec). In contrast, the mosquitoes in the recordings analysed here appeared proportionally bigger. Therefore small movements, like wing or leg movements, would often result in velocity readings of up to 10 cm/sec.

Since the purpose of this study was to measure the absolute contact times of mosquitoes towards the electrostatic netting, we chose the threshold to also include slow movements with continued contact with the netting (‘walking’). However, this specific behaviour was only rarely observed in the experiments performed here. We did therefore not distinguish between ‘sitting’ and ‘walking’ in our final analysis.

The video set-up presented here is not only usable in the laboratory, but also under field conditions. Compared to other equipment used to film mosquito behaviour, like large IR cameras [30] we used readily available and comparatively cheap, off-the shelf equipment costing around € 500 for one complete unit. The action cameras and their casings are rugged enough for the field, the equipment is versatile, and easy to maintain and repair. The cameras are being operated by off-the-shelf battery packs for cell phones or notebook computers, the LED-lights by 12V batteries. In our case, one battery pack of 12Ah could operate two cameras for one night and a 12V battery of 9Ah could operate the lighting equipment for up to six nights. During the time of our trials, we only had access to 64 Gb SD-cards, which limited the recording time to only 4.5 hrs per night. Additionally, in the field, cameras were set up in sleeping rooms occupied by residents and we wished not to disturb them in the middle of the night to change SD cards. We were therefore not able to use the video footage as a quantification method, e.g. numbers of mosquitoes visiting the eave tube during the complete night. However, SD-card capacities have now increased to 128 Gb or even higher, which translates into recording times of a minimum of nine hours. With that recording time it should be possible to also film mosquitoes that are active just before dawn.

Since we had to perform our observations under near-dark conditions with red LEDs as the single light source, the cameras had only a limited resolution. In order to obtain a sufficient contrast for the Ethovision software to be able to track the mosquito properly, the camera had to be placed close to the netting (max. 10 cm distance). With more light available, the camera will have a better resolution and can capture a wider area. For filming diurnal insects, for instance, one would have the option to either use the maximum angle of the camera or be closer to the objects, or use the minimum angle and film from further distance. It would also be possible to use several cameras and stitch the footage together using the Ethovision software.

## 5 Conclusions

We have shown that the behaviour of mosquitoes towards electrostatic eave tube netting, treated either with bendiocarb powder or with *B. bassiana* spores, results in sufficient contact to successfully transfer lethal doses to mosquitoes, both in the semi-field (with *Anopheles arabiensis*) as in the field. The videographic set-up used in this study is simple, sturdy and reliable enough to observe and analyse mosquito behaviour under field conditions.

## References

[1] World Health Organization. (2014). World Malaria Report 2014..

[2] Bhatt S, Weiss DJ, Cameron E, Bisanzio D (2015). The effect of malaria control on *Plasmodium falciparum* in Africa between 2000 and 2015.. Nature.

[3] Hemingway J (2014). The role of vector control in stopping the transmission of malaria: threats and opportunities.. Philos. Trans. R. Soc. Lond. B Biol. Sci..

[4] Edi CVA, Koudou BG, Jones CM, Weetman D (2012). Multiple-insecticide resistance in Anopheles gambiae mosquitoes, Southern Côte d’Ivoire.. Emerg. Infect. Dis..

[5] Cisse MBM, Keita C, Dicko A, Dengela D (2015). Characterizing the insecticide resistance of *Anopheles gambiae* in Mali.. Malar. J..

[6] Namountougou M, Simard F, Baldet T, Diabaté A (2012). Multiple insecticide resistance in *Anopheles gambiae s.l.* populations from Burkina Faso, West Africa.. PLoS One.

[7] Hiscox A, Khammanithong P, Kaul S, Sananikhom P (2013). Risk factors for mosquito house entry in the Lao PDR.. PLoS One.

[8] Bradley J, Rehman AM, Schwabe C, Vargas D (2013). Reduced prevalence of malaria infection in children living in houses with window screening or closed eaves on Bioko Island, Equatorial Guinea.. PLoS One.

[9] Ogoma SB, Lweitoijera DW, Ngonyani H, Furer B (2010). Screening mosquito house entry points as a potential method for integrated control of endophagic filariasis, arbovirus and malaria vectors.. PLoS Negl. Trop. Dis..

[10] Kirby MJ, Ameh D, Bottomley C, Green C (2009). Effect of two different house screening interventions on exposure to malaria vectors and on anaemia in children in The Gambia: a randomised controlled trial.. Lancet.

[11] Andriessen R, Snetselaar J, Suer RA, Osinga AJ (2015). Electrostatic coating enhances bioavailability of insecticides and breaks pyrethroid resistance in mosquitoes.. Proc. Natl. Acad. Sci. USA.

[12] Sternberg ED, Ng'habi KR, Lyimo IN, Kessy ST (2016). Eave tubes for malaria control in Africa: initial development and semi-field evaluations in Tanzania.. Malar. J..

[13] Snetselaar J, Njiru BN, Gachie B, Owigo P (in press). Eave tubes for malaria control in Africa: prototyping and evaluation against Anopheles gambiae s.s. and Anopheles arabiensis under semi-field conditions in western Kenya.. Malar. J.,.

[14] Sternberg ED, Waite JL, Thomas MB (2014). Evaluating the efficacy of biological and conventional insecticides with the new “MCD bottle” bioassay.. Malar. J..

[15] Vectorworks. Landscape of New Vector Control Products.. http://www.vector-works.org/resources/landscape-of-new-vector-control-products/.

[16] Ferguson HM, Ng’habi KR, Walder T, Kadungula D (2008). Establishment of a large semi-field system for experimental study of African malaria vector ecology and control in Tanzania.. Malar. J..

[17] Burkett DA, Butler JF (2005). Laboratory evaluation of colored light as an attractant for female *Aedes aegypti,*
*Aedes albopictus,*
*Anopheles quadrimaculatus,* and *Culex nigripalpus.*. Florida Entomol..

[18] Burkett DA, Butler JF, Kline DL (1998). Field evaluation of colored light-emitting diodes as attractants for woodland mosquitoes and other diptera in north central Florida.. J. Am. Mosq. Control Assoc..

[19] Gibson G (1995). A behavioural test of the sensitivity of a nocturnal mosquito, *Anopheles gambiae,* to dim white, red and infra-red light.. Physiol. Entomol..

[20] Takken W (2011). The role of olfaction in host-seeking of mosquitoes: A review.. Int. J. Trop. Insect Sci..

[21] Van Breugel F, Riffell J, Fairhall A, Dickinson MH (2015). Mosquitoes use vision to associate odor plumes with thermal targets.. Curr. Biol..

[22] Snetselaar J, Andriessen R, Suer RA, Osinga AJ (2014). Development and evaluation of a novel contamination device that targets multiple life-stages of *Aedes aegypti.*. Parasit. Vectors.

[23] Lwetoijera DW, Harris C, Kiware SS, Dongus S (2014). Increasing role of *Anopheles funestus* and *Anopheles arabiensis* in malaria transmission in the Kilombero Valley, Tanzania.. Malar. J..

[24] Yohannes M, Boelee E (2012). Early biting rhythm in the afrotropical vector of malaria, Anopheles arabiensis, and challenges for its control in Ethiopia.. Med. Vet. Entomol..

[25] Braack LEO, Coetzee M, Hunt RH, Biggs H (1994). Biting pattern and host-seeking behavior of *Anopheles arabiensis* (Diptera: Culicidae) in Northeastern South Africa.. J. Med. Entomol..

[26] Hammer Ø, Harper DAT, Ryan RD (2001). PAST: Paleontological statistics software package for education and data analysis.. Palaeontol. Electron..

[27] George J, Jenkins NE, Blanford S, Thomas MB (2013). Malaria mosquitoes attracted by fatal fungus.. PLoS One.

[28] Njie M, Dilger E, Lindsay SW, Kirby MJ (2009). Importance of eaves to house entry by anopheline, but not culicine, mosquitoes.. J. Med. Entomol..

[29] Kampango A, Bragança M, Sousa B, Charlwood J (2013). Netting barriers to prevent mosquito entry into houses in southern Mozambique: a pilot study.. Malar. J..

[30] Parker JE, Angarita-Jaimes N, Abe M, Towers CE (2015). Infrared video tracking of Anopheles gambiae at insecticide-treated bed nets reveals rapid decisive impact after brief localised net contact.. Sci. Rep..

